# Autophagy mediates pharmacological lifespan extension by spermidine and resveratrol

**DOI:** 10.18632/aging.100110

**Published:** 2009-12-23

**Authors:** Eugenia Morselli, Lorenzo Galluzzi, Oliver Kepp, Alfredo Criollo, Maria Chiara Maiuri, Nektarios Tavernarakis, Frank Madeo, Guido Kroemer

**Affiliations:** ^1^ INSERM, U848, F-94805 Villejuif, France; ^2^ Institut Gustave Roussy, F-94805 Villejuif, France; ^3^ Université Paris Sud-XI, F-94805 Villejuif, France; ^4^ Institute of Molecular Biology and Biotechnology, Foundation for Research and Technology, Heraklion GR-71110, Crete, Greece; ^5^ Institute of Molecular Biosciences, University of Graz, A-8010 Graz, Austria; ^6^ Equally contributed to this article

**Keywords:** AMPK, Caenorhabditis elegans, IKK, mTOR, p53, Saccharomyces cerevisiae

## Abstract

Although autophagy has widely been conceived as a self-destructive mechanism that causes cell death, accumulating evidence suggests that autophagy usually mediates cytoprotection, thereby avoiding the apoptotic or necrotic demise of stressed cells. Recent evidence produced by our groups demonstrates that autophagy is also involved in pharmacological manipulations that increase longevity. Exogenous supply of the polyamine spermidine can prolong the lifespan of (while inducing autophagy in) yeast, nematodes and flies. Similarly, resveratrol can trigger autophagy in cells from different organisms, extend lifespan in nematodes, and ameliorate the fitness of human cells undergoing metabolic stress. These beneficial effects are lost when essential autophagy modulators are genetically or pharmacologically inactivated, indicating that autophagy is required for the cytoprotective and/or anti-aging effects of spermidine and resveratrol. Genetic and functional studies indicate that spermidine inhibits histone acetylases, while resveratrol activates the histone deacetylase Sirtuin 1 to confer cytoprotection/longevity. Although it remains elusive whether the same histones (or perhaps other nuclear or cytoplasmic proteins) act as the downstream targets of spermidine and resveratrol, these results point to an essential role of protein hypoacetylation in autophagy control and in the regulation of longevity.

## Review

## Introduction

Autophagy (from the Greek, "auto" oneself, "phagy" to eat) involves the sequestration and degradation by lysosomal enzymes of old, supernumerary, damaged or ectopic organelles and/or portions of the cytoplasm [1]. At least three forms of autophagy have been described - macroautophagy, microautophagy, and chaperone-mediated autophagy - that differ with respect to the mode of cargo delivery to lysosomes [2,3]. This article will focus on macroautophagy (herein referred to as autophagy), the most important catabolic pathway that cells employ for the turnover of long-lived proteins and organelles and also one of the most prominent cytoprotective mechanisms in eukaryotic cell biology [4].

During macroautophagy, the cytoplasmic material targeted to degradation is delivered to lysosomes upon sequestration within double-membraned vesicles that are called autophagosomes. The generation of the autophagosome begins with the nucleation and elongation of the so-called phagophore, an isolation membrane that likewise originates from the endoplasmic reticulum. The edges of the phagophore then merge, resulting in the formation of a *bona fide* double-membraned autophagosome, which next fuses with a lysosome to generate an auto(phago)lysosome. Finally, the luminal content as well as the inner membrane of the auto(phago)lysosome (which together are known as "autophagic body") are degraded by lysosomal hydrolases. The end products of these catabolic reactions are exported to the cytoplasm, where they can re-enter anabolic and/or bioenergetic metabolisms [2,3,5,6].

The biochemical cascade that executes autophagy has originally been characterized at a molecular level in yeast (*Saccharomyces cerevisiae*) [7,8]. Hundreds of studies in different model organisms including mammals have confirmed that the essential machinery of autophagic sequestration and execution is phylo-genetically conserved, and hence involves the orthologs of a series of yeast genes that have been designated a ut ophag y-related (*ATG*) genes [7,8]. Autophagy likewise occurs at low baseline levels in all cells to ensure the homeostatic turnover of long-lived proteins and organelles [4]. Moreover, autophagy is upregulated well beyond basal levels: (i) when cells need to mobilize intracellular nutrients, as occurring during glucose and/or amino acid deprivation, hypoxia or growth factor withdrawal [2,3]; and (ii) when cells rid themselves of potentially noxious cytoplasmic materials including damaged organelles, aggregates of misfolded proteins, or invading microbes [9,10].

### The complex regulation of autophagy in response to stress

One of the key regulators of autophagy in human and murine cells is the mammalian target of rapamycin (mTOR, whose yeast ortholog is TOR) kinase, which suppresses autophagy in conditions of nutrient and growth factor repletion. Signal transducers including class I phoshatidylinositol-3-kinases (PI3Ks) and Akt link receptor tyrosine kinases to mTOR activation, thereby repressing autophagy in response to insulin, insulin-like growth factor (IGF) and other growth signals [11]. Activation of the mTOR complex 1 (mTORC1) - and consequent repression of autophagy - can also be mediated by mitogen-activated protein kinases (MAPKs) including extracellular signal-regulated kinases (ERKs) [12], by Ras-dependent activation of the p90 ribosomal S6 kinase [13], as well as by the Wnt signaling pathway [14]. Other prominent regulators of autophagy include (but are not limited to): AMP-activated protein kinase (AMPK), which inhibits mTOR in response to reduced ATP levels [15]; eukaryotic translation initiation factor 2α (eIF2α), which responds to nutrient deprivation as well as to double-stranded RNA (dsRNA) [16], ERN1 (whose yeast ortholog is known as IRE1), an endoplasmic reticulum (ER)-associated protein possessing intrinsic kinase and endoribonuclease activities and playing an important role in the alteration of gene expression upon ER stress [10,17]; and c-Jun N-terminal kinase (JNK), which is involved in multiple signaling cascades activated by stressful conditions [18].

Our own work in this field has added to this list of autophagy regulators: members of the Bcl-2 protein family that contain a single Bcl-2 homology (BH) domain, the so-called BH3-only proteins, which displace (and hence derepress) the essential autophagy modulator Beclin 1 from inhibitory complexes with Bcl-2 or Bcl-X_L_[19,20]; Sirtuin 1, which responds to high NAD^+^ levels, *de facto* acting as a sensor of nutrient availability [21]; the oncosuppressor protein p53, which inhibits autophagy when present in the cytoplasm [22]; the IκB kinase (IKK) complex, which is also essential for the activation of NF-κB by stress [23,24]; as well as the inositol 1,4,5-trisphosphate (IP_3_) receptor (IP_3_R) at the level of the ER [20,25]. Finally, autophagy is positively regulated by the transcription factor activity of E2F1 [26], FoxO3a [27,28], NF-κB [29] and p53 [30,31], among others. The apical events of the phylogenetically ancient molecular pathway for autophagy involve ULK1 and ULK2 (the mammalian orthologs of Atg1) as well as Beclin 1 (the human ortholog of Atg6). Beclin 1 functions as an allosteric activator of the class III PI3K hVps34 (which promotes phagophore nucleation/elongation via its product phosphatidylinositol-3-phosphate), and is part of a highly dynamic multiprotein complex that can incorporate various autophagic stimulators (*e.g.*, UVRAG, Bif-1/endophilin B1, Ambra 1) and/or inhibitors (*e.g.*, Bcl-2, RUBICON) [32-36].

**Figure 1.**
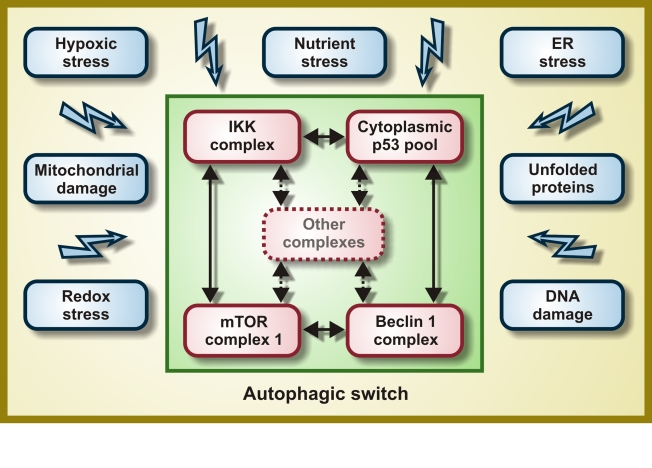
Molecular composition of the hypothetical autophagic switch. Irrespective of the primary stress signal, a homogeneous response would be obtained by the independent activation of molecular complexes organized around the IκB kinase (IKK), cytoplasmic p53, mTOR and Beclin 1. Within these complexes (and perhaps others), proteins would undergo reversible post-translational modifications and/or shuttle from one complex to another, thereby determining the function of the integrator/switch that activates autophagy. Please note that the autophagic switch is expected to contain several positive feedback loops that determine its (in)activation.

In synthesis, autophagy is connected to multiple stress pathways. In some cases, specific proteins and organelles are "tagged" for autophagic sequestration, implying that intrinsic features of the cargo determine its elimination by autophagy. This has been documented for proteotoxins [37-39], uncoupled mitochondria (a process that has been dubbed "mitophagy") [40-42], peroxisomes (for which the term "pexophagy" has been introduced) [43]; damaged ER (which is eliminated by "reticulophagy") [44]; and invading pathogens (which activate "xenophagy") [45]. In many other instances, autophagy occurs in a rather unselective fashion and represents a general response of the cell that transits from baseline to induced, yet limited levels. It is tempting to speculate that this change from basal to upregulated autophagy may involve the activation of a general "switch" that can respond to multiple distinct stress-responsive and damage-sensing pathways. Complex molecular switches regulate the clear-cut separation between discrete cellular states including the transition from an undifferentiated to a more differentiated state, the advancement of the cell cycle, or the "decision" to activate the apoptotic cascade [46,47]. Usually, such switches integrate diverse signals that are transmitted through negative feedback loops (which maintain homeostasis and keep cells in a defined state) and positive feedback loops (which mark the rapid evolution between two states) [48]. There is abundant evidence that the induction of autophagy involves positive feedback loops. For example, we have documented (i) that autophagy induced by rapamycin (which inhibits mTOR) is accompanied by the degradation of p53 and the activation of IKK; (ii) that pharmacological inhibition of p53 with pifithrin-α leads to mTOR inhibition and IKK activation; and that (iii) transgene-enforced activation of IKK stimulates p53 degradation at the same time as it inhibits mTOR [22-24,49]. This implies that mTOR inhibition, IKK activation and the degradation of cytoplasmic p53 are cross-linked through a network of self-amplifying feedforward loops (Figure 1), although it remains elusive how this occurs in molecular terms.

### Autophagy as a cytoprotective and anti-aging mechanism

Cells that are stressed and on the verge of death frequently manifest the cytoplasmic accumulation of autophagosomes and auto(phago)lysosomes, an observation that has been (mis)interpreted as if autophagy would contribute to the cellular suicide [50]. Thus, hundreds of papers have described "autophagic" (also dubbed "type 2") cell death, a cell demise subroutine preceded by massive autophagic vacuolization that is morphologically distinct from apoptosis ("type 1") and necrosis ("type 3") [51-54]. Although "autophagic cell death" undoubtedly exists as a morphological entity [53], this only exceptionally (at least in mammalian models) reflects the execution of cells by autophagy [50]. Rather, autophagy most frequently constitutes a (sometimes futile) mechanism of cellular adaptation to a diverse range of adverse conditions including hypoglycemia, hypoxia, lack of essential amino acids, absence of obligate growth factors or sublethal damage to cytoplasmic organelles including mitochondria and the ER [4,55,56]. Accordingly, the genetic inhibition of autophagy by knockout or knockdown of *ATG*genes often precipitates the apoptotic or necrotic death of cells that otherwise would survive nutrient depletion, growth factor withdrawal, hypoxia, ionizing radiation or anticancer chemotherapy [11,50,57-60]. Deficient autophagy is directly involved in a number of pathologies including neurodegenerative diseases, heart failure, hereditary myopathies, steatosis/steatohepatitis and other chronic inflammatory states [6,61-64]. Genetic and pharmacological manipulations designed to induce autophagy have been shown to protect cells against otherwise lethal damage *in vitro*[5,6]. Autophagyfavors the maintenance of high intracellular ATP levels [22,65], increases the capacity of cells to resist metabolic stress (hypoxia combined with nutrient deprivation) [22,66], prevents genomic instability [60,67] and limits the accumulation of potentially toxic proteins including proteotoxins that are responsible for neurodegeneration [10,38]. From a physiological point of view, aging can be viewed as a continuous decline in cellular and organismal functions that (at least partially) reflects the accumulation of misfolded proteins, oxidized lipids, as well as mutated mitochondrial and nuclear DNA.

The sole regimen leading to lifespan extension in every organism tested to date is dietary restriction, a reduction of the organism's caloric intake not associated to malnutrition [68]. Dietary restriction is a potent inducer of autophagy in virtually all species including mammals [69-71]. In the nematode *Caenorhabditis elegans*, autophagy is required for lifespan prolongation mediated by caloric restriction [72-74] or p53 depletion [22,49,75-77]. Thus, worms undergoing dietary restricttion do not live longer than control animals if concomitantly subjected to RNA interference (RNAi) against *atg* genes [72-74].

Rapamycin, which activates autophagy via inhibition of (m)TOR, has also been ascribed with prominent anti-aging properties, in various model organisms. However, rapamycin cannot extend the chronological lifespan (*i.e.*, the time post-mitotic cells survive during the stationary phase [78]) of yeast mutants that lack functional Atg1, Atg7 or Atg11 [79]. In *C. elegans*, the beneficial effects of rapamycin on longevity are lost when the essential autophagy modulator BEC-1 (the worm ortholog of mammalian Beclin 1 and yeast Atg6) is knocked down [74]. Thus, autophagy is required for rapamycin-mediated lifespan extension and delay of chronological aging in yeast and nematodes. Although it has not been formally demonstrated that rapamycin prolongs the lifespan of mice by inducing autophagy, even the treatment of pre-aged, genetically heterogeneous (out-bred) mice has been shown to increase longevity [80]. In mice, rapamycin avoids the age-related decline in hematopoietic stem cells function [81], an anti-senescence effect that has also been described *in vitro*[82,83].

Altogether, these results suggest that whole-body induction of autophagy by pharmacological agents may prolong the healthy lifespan, at least in laboratory conditions, supporting the idea that autophagy does not only confer cytoprotection but that it also has anti-aging effects at the organismal level.

### Autophagy mediates lifespan extension by resveratrol

Driven by the aforementioned considerations, we launched the working hypothesis that autophagy constitutes (one of) the major mechanism(s) through which longevity-extending drugs operate. We thus studied whether resveratrol, a well-studied anti-aging agent [84], would extend the lifespan of model organisms via the induction of autophagy. Although it also affects mitochondrial functions [85], resveratrol prominently acts as an allosteric activator of Sirtuin 1, a phylogenetically conserved deacetylase that senses the NAD^+^/NADH ratio [84]. Resveratrol increases the longevity of yeast, nematodes, and flies (*Drosophila melanogaster*) and also exerts anti-aging effects on mice kept on a high-fat diet [84,86]. Circumstantial evidence indicates that resveratrol can induce autophagy in yeast (although this was attributed to the oxidation of mitochondrial lipids [87]) and in human cancer cells (in which resveratrol-induced autophagy often precedes cell death [88]). Sirtuin 1 is the first protein that has been demonstrated to prolong lifespan in yeast (and then in animals including *C. elegans *and flies) [89], and has also been shown to trigger autophagy in human and murine cultured cells [90].

We confirmed that Sirtuin 1 overexpression increased the autophagic flux in human cancer cells *in vitro*, and that this effect was abolished by the addition of EX527, a pharmacological inhibitor of its catalytic activity [91,92]. Similarly, a transgene coding for SIR-2.1 (the *C. elegans *ortholog of human Sirtuin 1) caused autophagy in nematodes, suggesting that the link between Sirtuin 1 activation and autophagy is evolutionarily conserved [91,92]. Importantly, Sirtuin 1 was required for the induction of autophagy by nutrient deprivation (that was achieved by culturing cells in the absence of serum, amino acids and glucose) but not by other stimuli. Thus, in human cells, the depletion (by RNAi) or inhibition (with EX527) of Sirtuin 1 fully prevented the proautophagic effects of nutrient starvation, yet failed to affect the stimulation of autophagy by mTOR inhibition (with rapamycin), p53 inhibition (with pifithrin-α) or ER stress (triggered by the addition of tunicamycin). Similarly, loss-of-function mutations of *sir-2.1 *abolished autophagy induced by caloric restriction but not that promoted by rapamycin or tunicamycin in *C. elegans *[91,92]. Transgenic overexpression of *sir-2.1 *increased the median and maximum lifespan of nematodes as compared to non-transgenic control strains with the same genetic background. This gain in longevity was lost when the essential autophagic modulator BEC-1 was depleted by RNAi [91,92]. RNAi-mediated knockdown of the *C. elegans* p53 ortholog CEP-1, a manipulation that extends longevity through the stimulation of autophagy [77], failed to further ameliorate the beneficial effects of *sir-2.1 *overexpression on longevity [91,92]. This epistatic analysis suggests that SIR-2.1 accumulation and CEP-1 depletion extend lifespan through a common final pathway that relies on the induction of autophagy.

Another genetic intervention designed to indirectly activate Sirtuin 1 (or its worm ortholog SIR-2.1) consists in the transgenic overexpression of the gene coding for the pyrazinamidase/nicotinamidase PNC-1, which depletes nicotinamide, a negative regulator of Sirtuin 1/SIR-2.1. Transgenic overexpression of *pnc-1* did indeed induce autophagy in worms, and this response was abolished by RNAi-mediated depletion of SIR-2.1. Accordingly, the longevity-extending effects of PNC-1 were lost upon the knockdown of SIR-2.1, as well as upon that of either of the two essential autophagy modulators BEC-1 or ATG-5 [77]. Thus, both the overexpression and the metabolic activation of Sirtuin 1/SIR-2.1 increase lifespan through the induction of autophagy.

Next, we investigated whether resveratrol would induce autophagy in *C. elegans* via the activation of SIR-2.1. Addition of resveratrol to the worm culture medium did indeed stimulate autophagy, and this effect was lost upon RNAi-mediated depletion of SIR-2.1. Similarly, resveratrol reduced the aging-associated mortality of *C. elegans*, unless the products of *sir-2.1*or *bec-1 *were knocked down [77]. We concluded from these experiments that resveratrol prolongs lifespan in human and nematode cells by inducing autophagy, which results from resveratrol-mediated activation of Sirtuin 1/SIR-2.1 (rather than from an off-target effect).

### Autophagy mediates lifespan extension by spermidine

Driven by the fact that the intracellular level of polyamines declines in (otherwise healthy) aging humans [93], we investigated whether the polyamine spermidine display anti-aging properties. To address this question, we first took advantage of a yeast strain that is deficient in the ornithine decarboxylase SPE1, which catalyzes the first step of polyamine biosynthesis. In chronological aging experiments, *Δspe1* yeast cells exhibited an increased mortality (and hence a shortened lifespan), which could be restored to normal levels by supplementation with low doses (0.1 mM) of spermidine or its precursor putrescine [94]. Surprisingly, we found that higher concentrations of spermidine were able to increase the lifespan of wild type yeast cells with different genetic backgrounds. Thus, both chronological aging (which constitutes a model of post-mitotic aging) and replicative aging (which constitutes a model of stem cell aging) of yeast cells were significantly inhibited by spermidine supplementation. Lifespan prolongation in spermidine-treated yeast cells could be correlated with the reduced acetylation of several lysine residues located at the N-terminal tail of histone H3 (*i.e.*, Lys9, Lys14 and Lys18) [94]. Deletion of*sir2* (the yeast ortholog of *Sirtuin 1*) or any other sirtuin did not affect the ability of spermidine to extend chronological lifespan. Instead, epistatic analyses revealed that the anti-aging effect of spermidine was phenocopied by the knockout of histone acetylases, which hence were shown to regulate the same longevity-increasing pathway than spermidine does [94]. Moreover, spermidine efficiently inhibited general histone acetylase activity in extracts from purified yeast and mammalian nuclei in an *in vitro* assay [94]. These results suggest that spermidine acts differently from resveratrol. Thus, while the former inhibits histone acetylase(s), the latter stimulates the deacetylase activity of Sirtuin 1. However, formal evidence that the (de)acetylation of histones rather than that of other proteins (either in the nucleus or in the cytoplasm) account for the anti-aging properties of spermidine is still missing.

Microarray profiling of spermidine-treated yeast cells revealed the transcriptional activation of several autophagy genes including *atg7*,* atg11 *and *atg15*, and we indeed found that spermidine induces autophagy in yeast cells. Similarly, spermidine was highly efficient in upregulating the autophagic pathways when it was added to the culture medium or solid food of *C. elegans*or *D. melanogaster*, respectively. The same concentrations of spermidine that exerted pro-autophagic effects also had a marked lifespan-extending effect on yeast, nematodes and flies. The genetic inhibition of essential *ATG* genes (*i.e.*, knockout of *atg7* in yeast and flies, RNAi-mediated silencing of *bec-1* in nematodes) abrogated longevity extension induced by spermidine, indicating this polyamine can prolong lifespan by the induction of autophagy [94].

### Open questions

**Figure 2.**
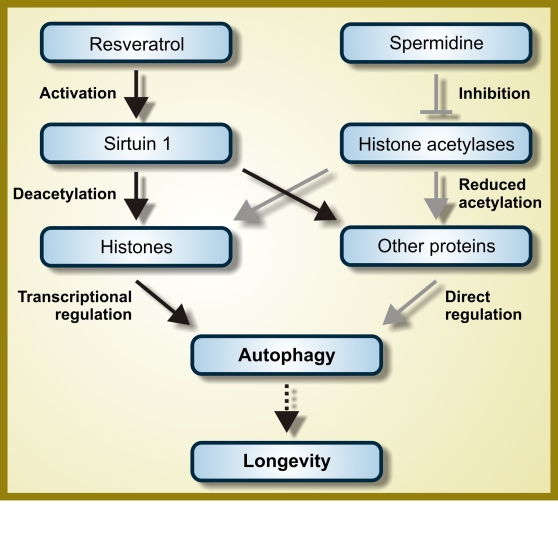
Hypothetical mode of action of resveratrol and spermidine as autophagy inducers. While resveratrol functions as an activator of the deacetylase Sirtuin 1, spermidine inhibits one or several histone acetylases. Therefore, both resveratrol and spermidine are expected to favor protein hypoacetylation. However, the autophagy-relevant substrates whose deacetylation is induced by resveratrol and spermidine are not fully characterized and it is even not known if they are completely distinct, partially overlapping or identical. For further details, please consult the main text.

The aforementioned results indicate that resveratrol and spermidine can prolong the lifespan of model organisms through the induction of autophagy (Figure 2). In addition, our work raises at least three issues that must be addressed by future investigation.

First, do resveratrol and spermidine extend longevity by acting on the same molecular pathway? While resveratrol can prolong lifespan through the activation of the deacetylase activity of Sirtuin 1 (or its non-mammalian equivalents SIR2 in yeast and SIR-2.1 in *C. elegans*), spermidine inhibits the general histone acetylase activity of yeast and mouse liver extracts. Clearly, histone (de)acetylation has been recognized as an important epigenetic regulator of longevity [95,96]. However, a fraction of Sirtuin 1 is present in the cytoplasm, from where it can directly deacetylate essential autophagic proteins (including ATG5, ATG7 and ATG8/LC3) [90], suggesting that (at least part of) the pro-autophagic effects of resveratrol derive from extranuclear, transcription-independent events. It will be important to know whether polyamines (like spermidine) and Sirtuin 1 activators (including resveratrol) can exert additive or synergistic effects on autophagy and longevity or whether these agents exactly activate the same molecular pathway. Moreover, the precise mechanisms by which spermidine and resveratrol control the autophagic switch awaits further exploration. Careful mechanistic and epistatic analyses are required to address this problem.

Second, do all longevity-prolonging manipulations induce autophagy? And is autophagy required for all such intervention to extend lifespan? Current results clearly indicate that autophagy is indispensable for the anti-aging action of rapamycin, resveratrol and spermidine. Moreover, it has been suggested that autophagy is required for longevity extension by dietary restriction in *C. elegans*, although this has not been tested for all caloric restriction protocols [73]. It remains an ongoing conundrum whether an increased level of autophagy is required in *C. elegans* for longevity extension conferred by the deficiency of GTPase RHEB-1 [97], the transcription faction hypoxia-inducible factor 1 (HIF-1) [98] and its negative regulator VHL-1 [99], the ubiquitin ligase WWP-1 [100], as well as the chaperones CCT4 and CCT6 [101]. A positive response to this question could establish a new paradigm in longevity research.

Third, and most important, can the data that we discuss here, which have mostly been obtained in simple model organisms and in laboratory conditions (where, for instance, the immunosuppressive side effects of resveratrol are certainly less incisive), be extrapolated to humans and to real life? Although rapamycin and polyamines can increase lifespan in mice [80,102], resveratrol only extends the longevity of mice that are kept on a high-caloric diet [86]. Clearly, rapamycin and resveratrol can induce autophagy *in vivo*, in mice [23,24,103]. However, it is thus far unknown whether there is indeed a cause-effect relationship between increased autophagy and healthy aging in mammals and in particular in humans. Such a causal relationship would revolutionize the entire field of aging research.

## Acknowledgments

NT is supported by grants from EMBO, the European Research Council (ERC) and the European Commission Coordination Action ENINET (contract number LSHM-CT-2005-19063). GK is supported by the Ligue Nationale contre le Cancer (Equipe labellisée), Agence Nationale pour la Recherche (ANR), European Commission (Apo-Sys, ChemoRes, ApopTrain, Active p53), Fondation pour la Recherche Médicale (FRM), Institut National du Cancer (INCa) and Cancéropôle Ile-de-France. EM is funded by a Ph.D. student grant from ApopTrain. LG is supported by the Apo-Sys consortium of the European Union. OK is funded by FRM.
